# The RaDiCo information system for rare disease cohorts

**DOI:** 10.1186/s13023-025-03629-z

**Published:** 2025-04-08

**Authors:** Paul Landais, Sonia Gueguen, Annick Clement, Serge Amselem, for the RaDiCo Program, Christine Bodemer, Patrick Calvas, Nicolas Chassaing, Annick Clement, Christian Corpechot, Vincent Cottin, Estelle Escudier, Bruno Fautrel, Sophie Georgin-Lavialle, Laurence Heidet, Bénédicte Héron, Xavier Jeunemaître, Bertrand Knebelmann, Agnès Linglart, Bernard Maitre, Jean-Louis Mandel, Irène Netchine, Aude Servais, Savine Vicart

**Affiliations:** 1 Childhood Genetic Diseases Laboratory, Université de Montpellier, Inserm U933, 26 rue Arnold Netter, 75012 Montpellier, France; 2 Chilhood Genetic Diseases Laboratory, Hôpital A. Trousseau, Inserm, Paris, France; 3 Chilhood Genetic Diseases Laboratory, Department of Paediatric Respiratory Medicine, Hôpital A. Trousseau, Assistance Publique Hôpitaux de Paris, Sorbonne Université, Inserm, Paris, France; 4 Chilhood Genetic Diseases Laboratory, Hôpital A. Trousseau, Sorbonne Université, Inserm, Paris, France

**Keywords:** Information system, Cloud computing, Infrastructure as a Service, Interoperability, GDPR, E-cohorts, Rare diseases, French Data Hub, European Research Network

## Abstract

**Background:**

Rare diseases (RDs) clinical care and research face several challenges. Patients are dispersed over large geographic areas, their number per disease is limited, just like the number of researchers involved. Current databases as well as biological collections, when existing, are generally local, of modest size, incomplete, of uneven quality, heterogeneous in format and content, and rarely accessible or standardised to support interoperability. Most disease phenotypes are complex corresponding to multi-systemic conditions, with insufficient interdisciplinary cooperation. Thus emerged the need to generate, within a coordinated, mutualised, secure and interoperable framework, high-quality data from national or international RD cohorts, based on deep phenotyping, including molecular analysis data, notably genotypic. The RaDiCo program objective was to create, under the umbrella of Inserm, a national operational platform dedicated to the development of RD e-cohorts. Its Information System (IS) is presented here.

**Material and methods:**

Constructed on the cloud computing principle, the RaDiCo platform was designed to promote mutualization and factorization of processes and services, for both clinical epidemiology support and IS. RaDiCo IS is based on an interoperability framework combining a unique RD identifier, data standardisation, FAIR principles, data exchange flows/processes and data security principles compliant with the European GDPR.

**Results:**

RaDiCo IS favours a secure, open-source web application in order to implement and manage online databases and give patients themselves the opportunity to collect their data. It ensures a continuous monitoring of data quality and consistency over time. RaDiCo IS proved to be efficient, currently hosting 13 e-cohorts, covering 67 distinct RDs. As of April 2024, 8063 patients were recruited from 180 specialised RD sites spread across the national territory.

**Discussion:**

The RaDiCo operational platform is equivalent to a national infrastructure. Its IS enables RD e-cohorts to be developed on a shared platform with no limit on size or number. Compliant with the GDPR, it is compatible with the French National Health Data Hub and can be extended to the RDs European Reference Networks (ERNs).

**Conclusion:**

RaDiCo provides a robust IS, compatible with the French Data Hub and RDs ERNs, integrated on a RD platform that enables e-cohorts creation, monitoring and analysis.

**Supplementary Information:**

The online version contains supplementary material available at 10.1186/s13023-025-03629-z.

## Background and significance

A disease is said to be rare when it affects fewer than 1 in 2000 individuals. Rare diseases (RDs) affect 26–30 million people in Europe and at least 3 million people in France. Around 70% of RDs begin in childhood [1]. RDs are often progressive, degenerative, and disabling, accounting for over a third of all deaths occurring in childhood. There is no treatment for most of them. They have a significant impact on patients’ quality of life, as well as on healthcare systems. RDs are mainly of genetic origin. Currently, 4913 genes have been implicated in 7532 different disease conditions [2].

In France, three consecutive national RD plans (i.e., “PNMR” 1, 2 and 3, standing for Plan National Maladies Rares 1, 2 and 3) have structured the healthcare offering [3]. They rely on 23 RD Healthcare Networks (RDHNs), whose topics are like those covered by the 23 ERNs, and represent 2241 accredited sites throughout the country. A National Rare Disease Data Bank [4], the BNDMR [5], registers RD patients based on a unique RD identifier [6] and a minimum data set [7]. Thus, nearly 1.5 million patients and 5700 distinct diagnoses have already been identified.

RDs clinical care and research face several challenges. The number of patients per disease is limited. Patients are dispersed over large geographic areas, making data collection difficult. For a given RD, the number of researchers involved is very limited. Current databases as well as biological collections, when existing, are generally local, of modest size, incomplete, of uneven quality, heterogeneous in format and content, and rarely accessible or standardised to support interoperability. For many RDs, the disease phenotypes are complex and correspond to multi-systemic conditions; they are partially described over time, with insufficient interdisciplinary cooperation.

Thus emerged the need to generate, within a coordinated, mutualised, secure and interoperable framework, high-quality data from national or international RD cohorts, based on deep phenotyping and including molecular analysis data, notably genotypic.

The RaDiCo program, coordinated by Inserm, has been funded by the National Research Agency for the 2011–2024 period in the framework of the “Investments for the Future” program. It was dedicated to creating of a national operational platform to support RD e-cohorts selected from a national call.

To support the RD cohorts electronically, the RaDiCo platform was built with the complementary expertise of two teams: Clinical Epidemiology Research, and e-Health & Information Technology, respectively. In a previous article, we described the data related to the secondary objectives of the cohorts [8]. Here, we describe the RaDiCo IS and its specifications to support epidemiological research on RD e-cohorts.

## Materials and methods

To develop the RaDiCo IS, we first defined several principles and steps (Table 1).

**Table 1:** RaDiCo IS implementation steps

1) Identifying the RD ecosystem and related actors	
2) Designing the functional architecture allowing access to the platform	
3) Capturing medical data through an electronic Clinical Report Form (e-CRF)	
4) Developing an Infrastructure as a Service (IaaS) in the cloud	
5) Conceiving a « secure by design» infrastructure	
6) Developing applications that will allow data exchange and security	
7) Developing security procedures compliant with the European General Data Protection Regulation (GDPR)	
8) Conceiving the global data architecture, which articulates its different components	

To achieve semantically interoperable data exchange, we used controlled terminology standards, the Orphanet terminology for RDs [9] and the Medical Dictionary for Regulatory Activities [10]. To support the interoperability, we standardised the data and used ontologies, Human Phenotype ontology [11] and Orphanet Rare Disease Ontology [12]. Exchange format standards specify the way information is encoded. We used SAS statistical software, R formats and Extensible Markup Language (xml). The standards of the Clinical Data Interchange Standards Consortium (CDISC) [13] were proposed by REDCap [14]. We also complied to the FAIR principles [15]. The cohorts’ implementation followed the STROBE recommendations [16].

### The RD ecosystem

**Fig. 1:**
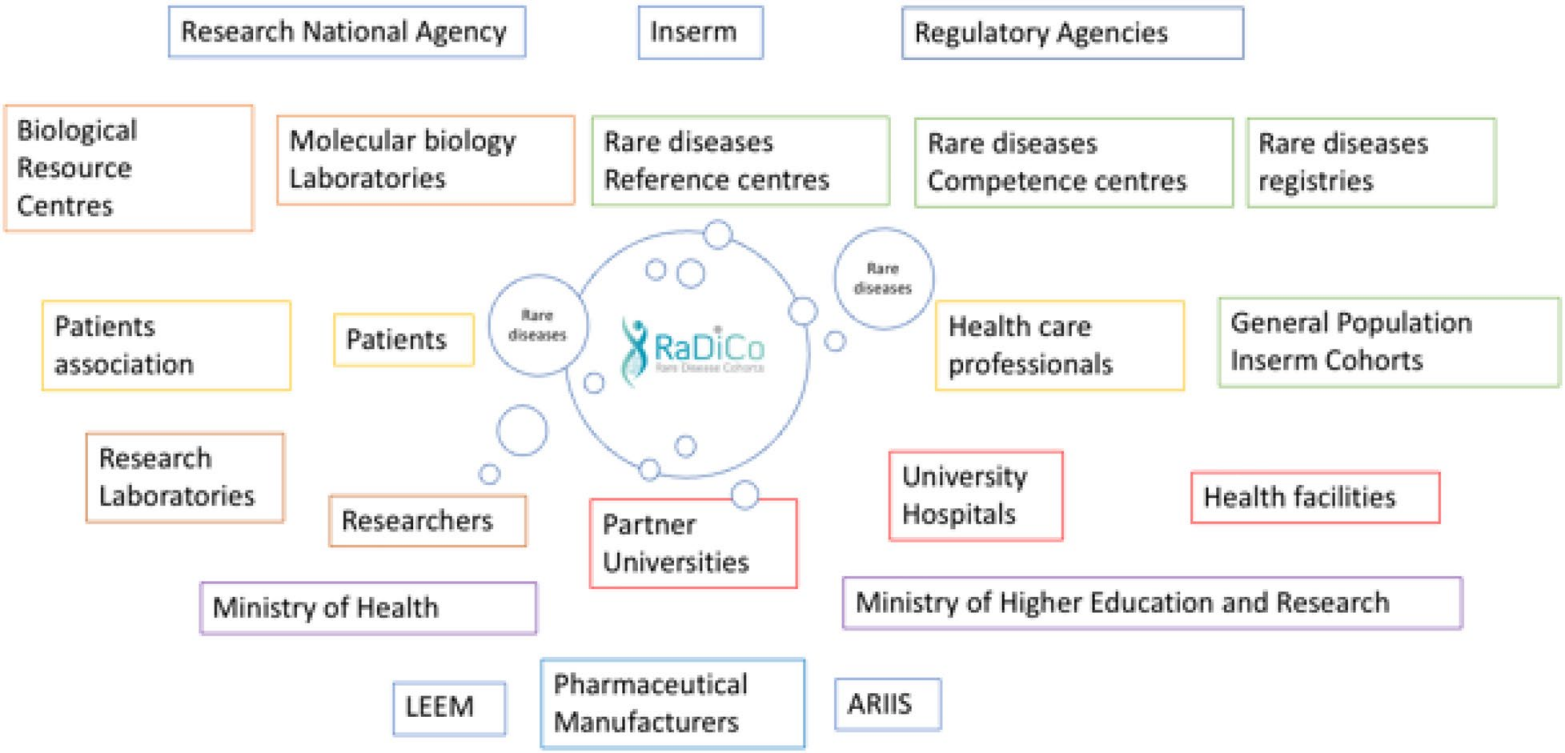
RaDiCo and its Rare Disease ecosystem. (LEEM: The drug companies; ARIIS: Health Industry Alliance for Research and Innovation)

RaDiCo operates in a complex ecosystem with a large number of actors. For its 13 cohorts, RaDiCo is linked to 180 RDs centers, due to the low number of patients per RD and the geographic dispersion of patients throughout France. Figure 1 describes the actors involved in the program. Identifying the plurality of the protagonists as well as their job specificities enables defining the structure of the information demand, as well as the IS needs and the interconnections to develop.

### Management of the RaDiCo program

The overall management of the RaDiCo program is provided by an executive committee, a scientific committee, and an institutional committee. In addition, each cohort has its own governance structure, with its steering and scientific committees.

Before their implementation, cohort projects were all submitted to ethical and regulatory authorities and to the French Data Protection Agency. The RaDiCo executive committee that organises a mutualised administrative monitoring, ensures contacts with the National Research Agency, Inserm, ministerial authorities, universities, and industrial partners. Cohort implementation and development are planned on a cohort-by-cohort basis; this includes cohort design, identification of key milestones, implementation, and monitoring meetings. Over 50% of cohort funding is provided by industrial and academic contracts. Human resources management is carried out centrally.

## Results

### Drafting the e-cohorts Case Report Forms (CRFs)

The research questions for a given cohort are relayed by the eCRF produced using the REDCap software [17]. A Data Monitoring Plan integrates the monitoring checklist and defines the modalities and frequency of visits, the nature of the data and the percentage of files to be monitored. Thereafter, a Data Validation Plan is elaborated to organise and prepare the elements required for data management. The Data Management Plan is then designed to check whether the cohort is adequately meeting its objectives: compliance with the monitoring schedule, number of patients recruited, data completion rate, number of data verified, compliance with defined Key Performance Indicators (KPIs). A RACI (Responsible, Accountable, Consulted, Informed) matrix of the roles and tasks of each RaDiCo team member is summarised in Table 2. A quality committee meets every two weeks to monitor compliance with the platform’s standardized operating procedures (SOPs).

**Table 2:** RACI matrix of the different steps of e-CRF implementation

Task roles	CRF	Annotated CRF	e-CRF conception	Entry guide	e-CRF testing	Monitoring plan	Validation plan	Data-management plan	Quality steering committee
PI	C	I	I	C	C	C	C		
O&S Director	A	A	A	I	I	A	A	A	A
Clin Res PM	R	C	C	R	R	I	I	R	R
E-Health PM	C	C	C	C	C	C	C	R	R
IS Manager	I	I	I	I	I	C	C	I	I
CRTs	C	I	I	C	C		C		R
CRAs	C	I	I	C	C	C			R
Data-manager	C	R	R	A	A	R	R	R	R
Biostatistician	C	C	C	I	C	I	I	C	

R: Responsible; A: Accountable; C: Consulted; I: Informed, PI: Principal Investigator; O&S Director: Operational and Scientific Director; Clin Res PM: Clinical Research Project Manager; E-Health PM: Electronic-health Project Manager; IS Manager: Information System Manager; CRTs: Clinical Research Technicians; CRAs: Clinical Research Assistants

### Translating the clinical research needs in a capability map.

**Fig. 2:**
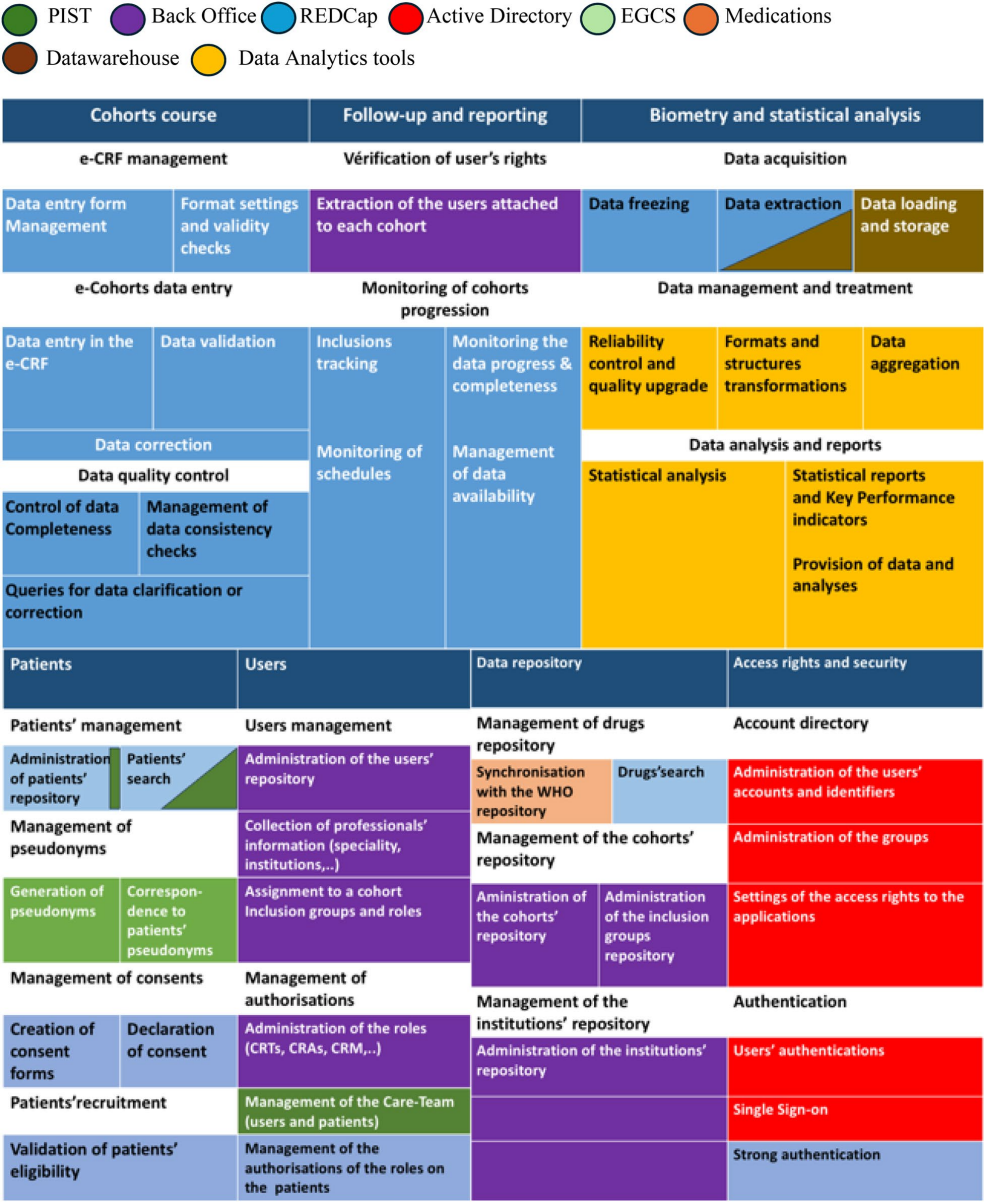
RaDiCo Capability map and the corresponding RaDiCo applications.  PIST,  Back Office,  REDCap,  Active Directory,  EGCS,  Medications,  Datawarehouse,  Data Analytics tools

Applications designed to meet the needs of clinical research are summarised in the RaDiCo capability map (Fig. 2). This map characterises the progress of the clinical study course, the monitoring and reporting phase, the statistical analyses and reporting of results, the management of patients and professionals' data, of data repositories, access rights and security*.*

### Planning the design of the information system (IS)

According to this map, the stages in building the IS were thus designed as follows:

- Definition of the IS needs.
- Drafting of IS specifications.
- IS development including the integration of the eCRF into the IS.
- Preparation of methods and tools.
- IS validation.
- Tests and recipes.
- Production of the first version of the IS, and thereafter of its versioning, stored in Github.

### Software architecture and applications

#### Functionalities and users

The RaDiCo IS allows the management of data repositories, the collection of patient data (personal and medical) and the performance of studies linked to the data collected. It hosts data linked to two types of profiles, i.e., users and patients. On the one hand, IS users are either healthcare personnel, research professionals or computer scientists, involved in data collection, formatting and processing, as well as in IS maintenance. On the other hand, the patients represent the source of data collection and may also enter themselves questionnaires such as those on quality of life or data through medical devices, if any.

The RaDiCo IS has been conceived on the principle of an Infrastructure as a Service (IaaS), a cloud computing model. The platform manages servers' middleware, application software (executables, settings), integration of a service-oriented architecture (SOA), and databases. The cloud provider, either Microsoft Azure or CompuGroup Medical in this case, manages the server hardware, virtualization layers, storage, and networks.

#### A three-tenant architecture

**Fig. 3:**
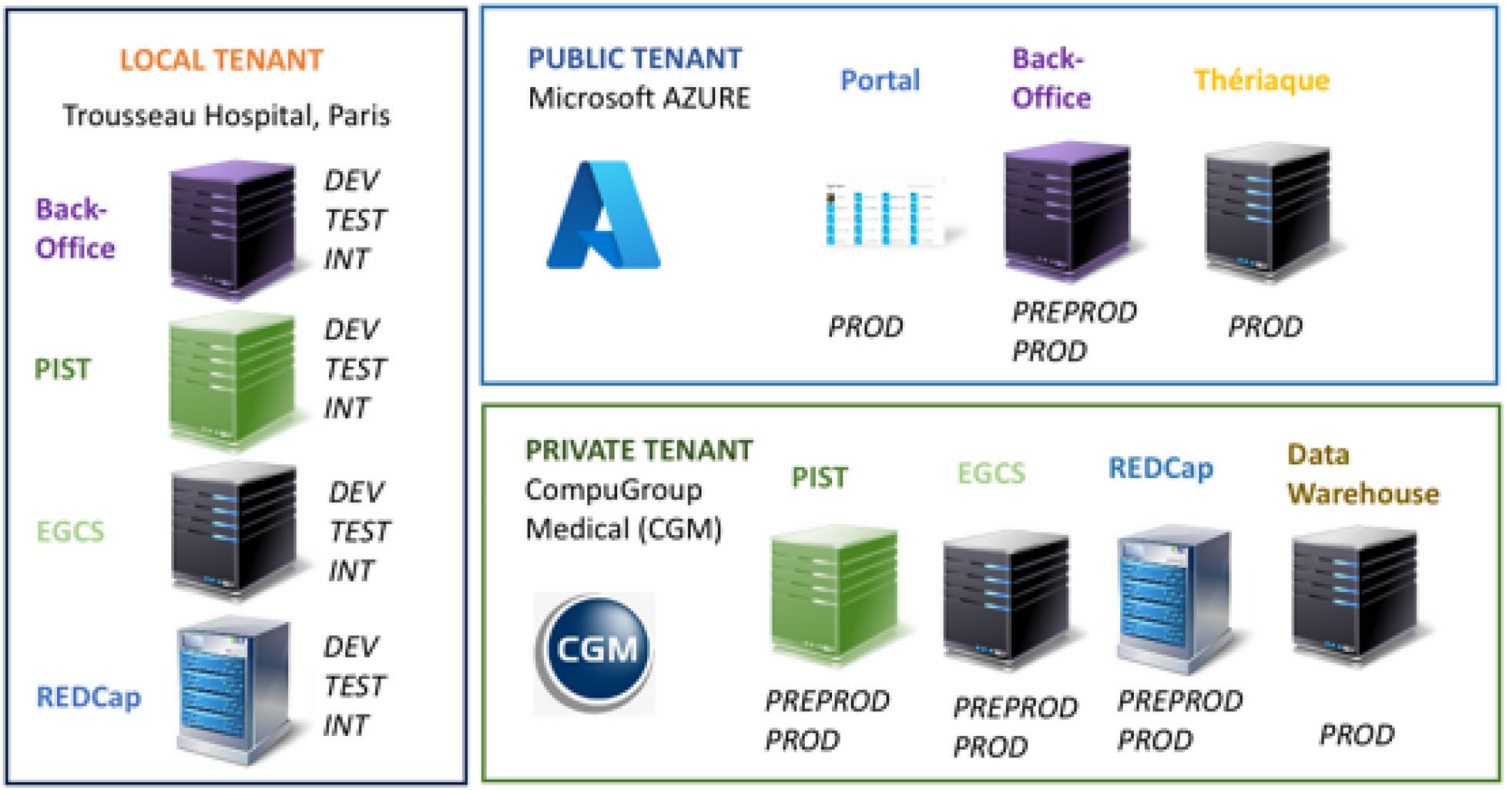
RaDiCo SI, a three tenant architecture. (Development: DEV; Tests: TEST; Integration: INT; Pre-production: PREPROD; Production: PROD)

The RaDiCo IS architecture is distributed over 3 tenants (Fig. 3).

- *The local tenant* corresponds to the on-premises infrastructure located within the Trousseau hospital, Paris, France. It hosts the non-production environments for development, integration and testing of RaDiCo IS applications.
- *The public cloud tenant* corresponds to the infrastructure hosted and managed by the Microsoft Azure provider. It hosts all the applications that do not have any patient-related information.
- *The HDS tenant* corresponds to a private Health Data Hosting (HDS) environment approved by the French Digital Agency for Health (https//esante.gouv.fr), managed by the CompuGroup Medical (CGM) company. It hosts all the applications that contain patient-related data (personal or medical data).

#### RaDiCo IS applications

The RaDiCo IS is made up of a set of seven applications appearing in the capability map, covering the required functionalities that are distributed across the different tenants. These applications are as follows.

- Portal and Azure Active Directory (AD)

The RaDiCo portal is a « software as a service» application provided by Microsoft on the Azure platform. It authenticates users and adapt the display of the applications that users can access to. Access rights are configured on the RaDiCo AD, and synchronised with the Azure AD.

- Back-office (BO)

The BO application includes repositories for RaDiCo IS users, cohorts, inclusion groups, institutions, as well as their specific roles. It does not contain medical or nominative data. The BO manages user accounts by controlling the users’ functional authorizations with respect to the cohorts and inclusion groups they are concerned with, according to a rights matrix. An inclusion group links patients and users, regardless of their geographical place of practice. The BO also includes a rights matrix related to medical data in REDCap (see below).

- The Patient Identifier System and Translation (PIST)

The PIST application, designed by RaDiCo, is the patients’ repository. It stores three important pieces of information: Patient identifiers, the Global Unique Identifier (GUID), and the unique RD identifier (IdMR) [6], the patient’s RD pseudonym, which gives access to medical data, patients’ personal data and to the care-team. A care-team lists all healthcare professionals authorised to access data of patients they care. PIST does not host medical data. This application is built on a “Security by design” logic, separating personal data from medical information stored in REDCap. Stored data are encrypted for and within the database.

- Electronic Gateway Controller Service (EGCS)

The EGCS orchestrates the patient creation/search processes between the different applications. It links the GUID code, the RD identifier and the RaDiCo code (see below). These lists are hosted within the EGCS in the form of a cross reference between the codes of each patient and the users according to their specific rights.

- Thériaque

It is the repository of medicines available in France, synchronised with the International Non-proprietary Names of the WHO [18]. It standardises medical treatments coded in REDCap.

- REDCap

It is a secure, web-based application [14] from the Vanderbilt University, USA. It has been designed to support data capture for research studies. REDCap contains all the medical data of patients included and followed in the RaDiCo cohorts. A RaDiCo code for each patient is associated with the medical data. Access to PIST and EGCS is possible via an extension module called “hook” that allows the execution of a code or a program. Audit trails are available to track data manipulations. Automated export procedures for seamless data downloads are possible towards common statistical packages. Procedures for importing data from external sources are also available. REDCap does not host nominative data.

- Datawarehouse (DWH)

The RaDiCo DWH is a relational database hosted in the private cloud. It collects data from the cohorts with the primary objective of data analysis. Like all DWHs, it is defined as a set of subject-oriented, integrated, time-varying and non-volatile data. Stored data are not nominative.

### Data architecture

The macroscopic data model of the RaDiCo IS, associated with its applications, can be broken down according to its functional entities:

- e-cohorts for clinical studies.
- Professionals’ roles on the RaDiCo platform.
- Institutions with users place of practice.
- Inclusion groups that link patients and users, regardless of their geographical place of practice.
- Users of RaDiCo IS.
- Groups characterised by the authorised groups configured in the AD.
- Care-teams: all users authorised to view or intervene on patients' data.
- Patients' nominative data.
- Patients' medical data.
- Medications.

**Fig. 4:**
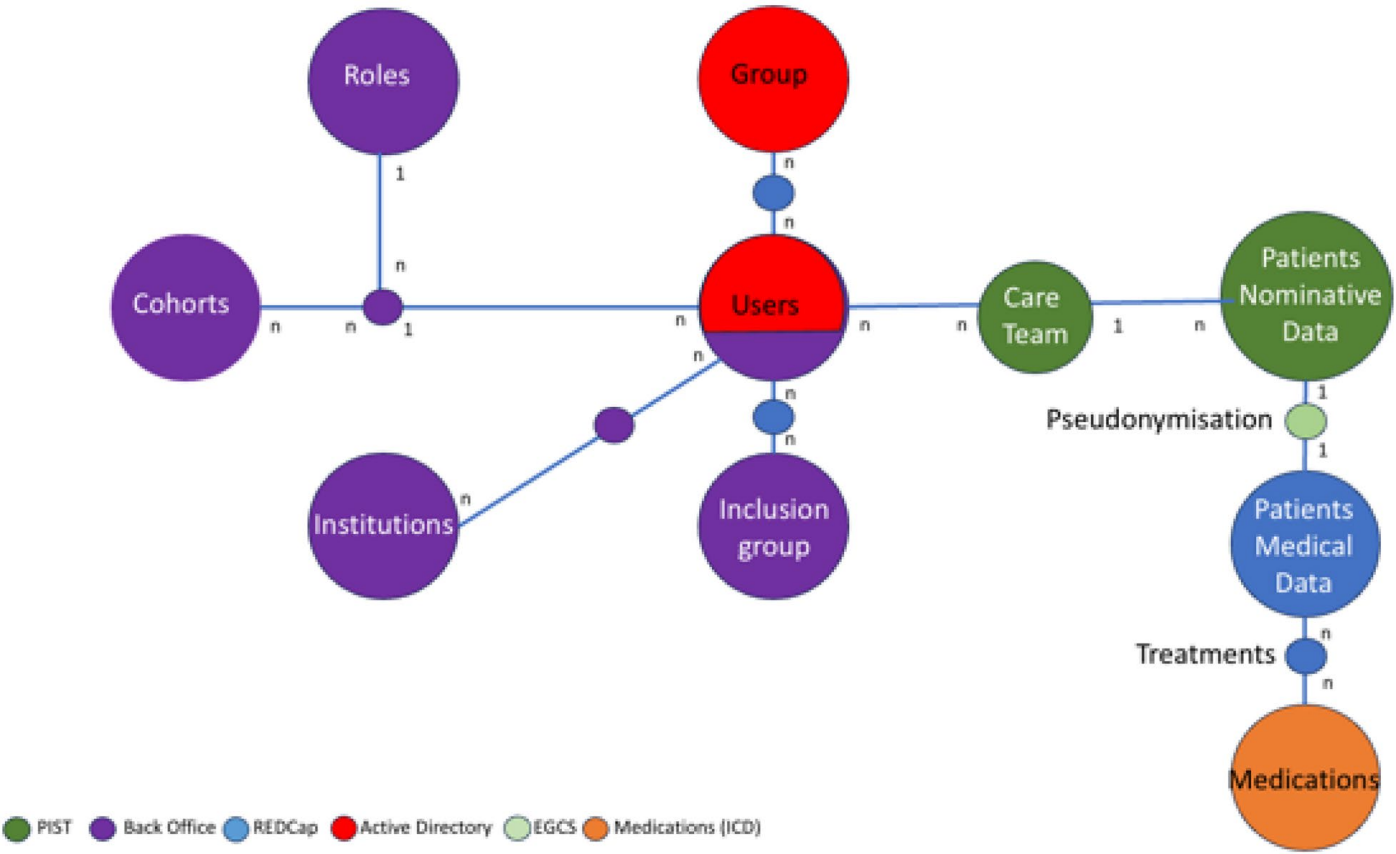
RaDiCo data model representing links between functional entities and dedicated applications

**Fig. 5:**
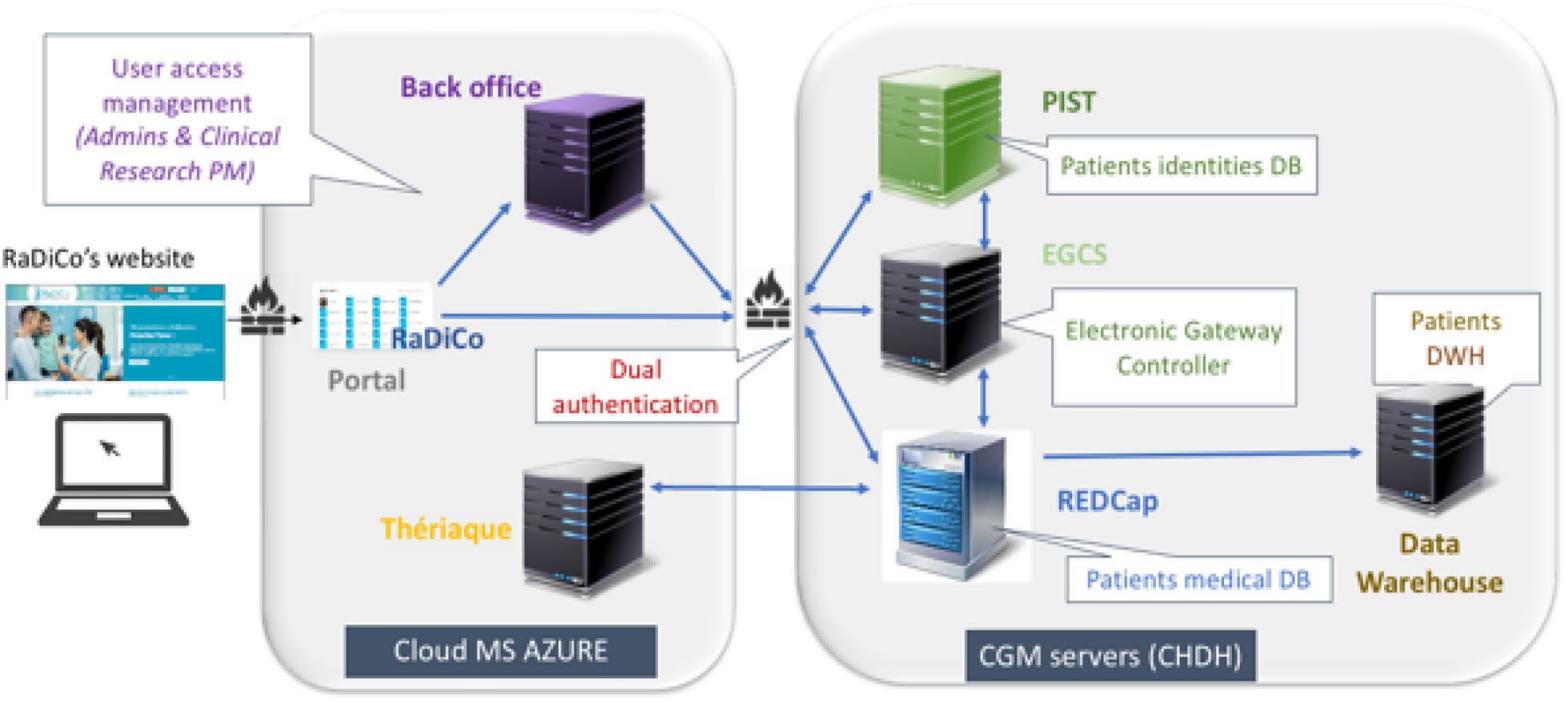
RaDiCo Information System. (Cloud Microsoft AZURE; CHDH: Certified Health Data Host; CGM: CompuGroup Medical; Portal: Users authentication & selected access to the cohorts; Back Office: Management of access rights; PIST: Patient Identification System Translator; EGCS: Electronic data capture Gateway Controller Service; REDCap: Research Electronic Data Capture; DWH: RaDiCo Data warehouse; Thériaque: Drugs databank)

The general data architecture is modelled as depicted in Fig. 4. Most importantly, the security and anonymity of patient information are ensured by segregation between a patient's nominative data (hosted in PIST only) and their medical data (hosted in REDCap only), thereby defining a security by design. The overall architecture of the RaDiCo IS appears on Fig. 5.

### Security

The security of the RaDiCo IS is based on a certified HDS, a strong and multi-factor authentication system, the fine-tuned management of access rights and digital signature mechanisms for data leaving the IS. Security mechanisms provide access control to all environments through the Azure portal application. A second security is added for access to the production environments of the HDS environment via the multi-factor authentication. Personal and medical data are hosted in the private HDS tenant. Access to patient data is authorised according to a double correspondence: it is on the one hand the authorisation of access to patients modelled in the care-teams, and on the other hand the qualification of the user's effective rights, modelled in the rights matrix at the BO and REDCap levels.

### Maintenance in operational condition (MCO)

Backups are carried out on a regular basis. The number of environments is suitable for software development, integration, staging and production. The GitHub tool is used to manage software configuration and code versions.

### The RaDiCo cohorts

Inclusion of prevalent and incident cases started at the end of 2016 after all scientific, legal and regulatory formalities had been completed. This preparation stage was particularly long due to the French cumbersome procedures, which since then have been simplified. As of March 2024, 8063 patients have been included within the 13 RD e-cohorts, covering 67 RDs (Table 3). The principal investigators (PIs) of these cohorts belong to 10 RD European Reference Networks [19]. They contributed to the European Joint Program on RDs (EJP-RD) [20], which recently evolved into the ERDERA [21] partnership (European Rare Diseases Research Alliance).

**Table 3:** The 13 RaDiCo cohorts by alphabetic order of their acronyms, with the names of their Rare Disease Healthcare Network (RDHN) and their contribution to RDs European Research Networks (ERNs)

Cohorts’ acronyms	Diseases	RDHN	ERN
AC-OEIL	Congenital Eye defects	SENSGENE AnDDI-Rares	ERN-Eye
AcoStill	Still’s disease	FAI2R	ERN-Rita
COLPAC	Low Phospholipid-Associated Cholelithiasis syndrome	FILFOIE	
DCP	Primary Ciliary Dyskinesia	RESPIFIL	ERN-Lung
ECYSCO	Cystinosis	ORKiD	ERKnet
EURBIO-Alport	Alport Syndrome	ORKiD	ERKnet
FARD	Rare Skin Disease Burden	FIMARAD	ERN Skin
GenIDA	Intellectual deficiency and autism spectrum disorders	DéfiScience	
IDMet	Imprinting Disorders	FIRENDO OSCAR DéfiScience	ENDOERN
MPS	Mucopolysaccharidoses	G2M	MetabERN
PID	Idiopathic Interstitial Pneumonia	RESPIFIL	ERN-Lung
PP	Periodic Paralysis	FILNEMUS	Euro-NMD
SEDVasc	Vascular Ehlers-Danlos	FAVA-Multi	VASCERN

Many original results have been obtained in relation with the secondary objectives of the RaDiCo cohorts (Table 4). The data in relation with the primary objectives are currently under analysis.

**Table 4:** International publications so far associated with the RaDiCo cohorts classified by main topics

Discovery of new disease genes	TTC12 loss-of function mutations cause primary ciliary dyskinesia and unveil distinct dynein assembly in motile cilia vs. flagella [22] Lack of GAS2L2 causes primary ciliary dyskinesia by impairing cilia orientation and mucociliary clearance [23] Mutations in outer dynein arm heavy chain DNAH9 cause motile cilia defects and situs Inversus [24] Mutations in DNAJB13, encoding an HSP40 family member, cause primary ciliary dyskinesia and male infertility [25] de novo missense variants in FBXW11, a gene that encodes an F-box protein involved in ubiquitination and proteosomal degradation [26] Bi-allelic variants in WNT7B disrupt the development of multiple organs in humans [27] First evidence of SOX2 mutations in Peter’s anomaly: lessons from molecular screening of 95 patients [28] Evaluation of somatic and/or germline mosaicism in congenital malformation of the eye [29] Mutations in DNAH17, Encoding a Sperm-Specific Axonemal Outer Dynein Arm Heavy Chain, Cause Isolated Male Infertility Due to Asthenozoospermia [30]
Disease management treatment assessment and prognosis	Vascular Ehlers-Danlos syndrome – Long-term observational study [31] An international cohort study spanning five decades assessed outcomes of nephropatic Cystinosis [32] Expectations about treatment of idiopathic pulmonary fibrosis: comparative survey of patients, carers, and physicians (the RESPIRATORY French study) [33] Follow-up and management of chronic rhinosinusitis in adults with primary ciliary dyskinesia: review and experience of our reference centers [34] Growth Patterns and Outcomes of Growth Hormone Therapy in Patients with Acrodysostosis [35] Height and Body Mass Index in Molecularly Confirmed Silver-Russell Syndrome and the Long-Term Effects of Growth Hormone Treatment [36] Impact of a Rare Respiratory Diseases Reference Centre Set-up on Primary Ciliary Dyskinesia Care Pathway [37] Obeticholic Acid as a Second-Line Treatment for Low Phospholipid-Associated Cholelithiasis Syndrome [38] Prevalence and Course of Disease after Lung Resection in Primary Ciliary Dyskinesia: A Cohort & Nested Case–Control Study [39] Recombinant Growth Hormone Improves Growth and Adult Height in Patients with Maternal Inactivating GNAS Mutations [40] Spontaneous Cervical Artery Dissection in Vascular Ehlers-Danlos Syndrome: A Cohort Study [41] The Impact of Lockdown on Young People with Genetic Neurodevelopmental Disabilities: A Study with the International Participatory Database GenIDA [42] Treatment of Idiopathic Pulmonary Fibrosis with Capsule or Tablet Formulations of Pirfenidone in the Real-Life French RaDiCo-ILD Cohort [43]
Pathophysiology, diagnostic and clinical approaches	Accuracy of clinical diagnostic criteria for patients with vascular Ehlers-Danlos syndrome in a tertiary referral centre [44] Functional assessment and phenotypic heterogeneity of SFTPA1 and SFTPA2 mutations in interstitial lung diseases and lung cancer [45] Pulmonary fibrosis in children [46] Chronic interstitial lung diseases in children: diagnosis approaches [47] Pulmonary hemosiderosis in children with Down syndrome: a national experience [48] Paediatric sarcoidosis [49] Genetic causes and clinical management of pediatric interstitial lung diseases [50] Assessment of arterial damage in vascular Ehlers-Danlos syndrome: a retrospective multi centre cohort [51] Clinical and functional heterogeneity associated with the disruption of retinoic receptor beta [52] Clinical, genetic and biochemical signatures of RBP4-related ocular malformations [53] Impact of Gender on the Characteristics of Patients with Idiopathic Pulmonary Fibrosis Included in the RaDiCo-ILD Cohort [54] Low-Phospholipid-Associated Cholelithiasis Syndrome: Prevalence, Clinical Features, and Comorbidities [55] Neonatal and Early Infancy Features of Patients With Inactivating PTH/PTHrP Signaling Disorders/Pseudohypoparathyroidism [56] Otological Manifestations in Adults with Primary Ciliary Dyskinesia: A Controlled Radio-Clinical Study [57] Standardised Clinical Data from Patients with Primary Ciliary Dyskinesia: FOLLOW-PCD [58] Incontinentia pigmenti burden scale [59] Burden of albinism: development and validation of a burden assessment tool [60] Burden of adult neurofibromatosis 1: burden assessment tool [61] Health-related quality of life in infants and children with interstitial lung disease [62]
Genotype–phenotype relationship	Infertility in an adult cohort with primary ciliary dyskinesia: phenotype-gene association [63] Primary ciliary dyskinesia gene contribution in Tunisia: Identification of a major Mediterranean allele [64] Alport syndrome: a unified classification of genetic disorders of collagen IV α345 [65] Genetics of anophthalmia and microphthalmia. Part 1: Non-syndromic anophthalmia/ microphthalmia [66] Deep phenotyping, including quantitative ciliary beating parameters, and extensive genotyping in primary ciliary dyskinesia [67] Evaluation of somatic and/or germ line mosaicism in congenital malformation of the eye [68] Frequency of de novo variants and parental mosaicism in vascular Ehlers-Danlos syndrome [69] High Nasal Nitric Oxide, Cilia Analyses, and Genotypes in a Retrospective Cohort of Children with Primary Ciliary Dyskinesia [70] Motile Cilia and Airway Disease [71] Topological data analysis reveals genotype–phenotype relationships in primary ciliary dyskinesia [72]
Methodological aspects	Federating patients identities: the case of rare diseases [6] Cerberus, an access control scheme for enforcing least privilege in patient cohort study platforms [73] National registries of rare diseases in Europe: an overview of current situation and experiences [74] Recommendations for improving the quality of rare disease registries [75] Data quality in rare diseases registries [76] GenIDA, a Participatory Patient Registry for Genetic Forms of Intellectual Disability Provides Detailed Caregiver-Reported Information on 237 Individuals with Koolen-de Vries Syndrome [77] GenIDA: An International Participatory Database to Gain Knowledge on Health Issues Related to Genetic Forms of Neurodevelopmental Disorders [78] Registries and Collaborative Studies for Primary Ciliary Dyskinesia in Europe [79] The Disease-Specific Clinical Trial Network for Primary Ciliary Dyskinesia: PCD-CTN [80]

## Discussion

The RaDiCo IS was designed to support RD e-cohorts with innovative and general principles including factorization, pooling of resources, tools, skills and know-how, industrialization of processes, as well as quality control and security. Several implementation criteria were retained: quality, security, scalability, sustainability, and cost-efficiency.

The goal of the platform is dual. On one side, it offers a technical and IS support to maintain the running cohorts and answer to the questions defined by the research protocol. On the other side it enables running new cohorts projects. The technical support brought by the platform and its IS are structures dedicated to last within the institutional framework of Inserm. The infrastructure may be shared and pooled at the European or international level according to needs.

We have set up an e-Health & IS team to develop modern means of constructing e-cohorts with security controls supported by professionals in the field. Software and technical developments were carried out with service providers. As detailed above, the architecture includes several original modules designed by RaDiCo: PIST, Back Office and EGCS, all upstream of REDCap.

Other significant advances include the use of the unique and non-reversible RD identifier we have developed, the use of webservices, the possibility for patients to enter self-questionnaires, or to connect mobile objects.

The RaDiCo IS uses standards that favour interoperability of data and services, including the use of ontologies and FAIR principles. It complies with the European directive on the GDPR. Of note, the separation of the applications between the three tenants was chosen for cost reasons because HDS, with equal characteristics, costs around four times as much as public cloud hosting on Microsoft Azure. The data are hosted by an approved HDS (CompuGroup Medical, CGM), complying with 4 major criteria: availability, confidentiality, integrity, and traceability of the data. An original feature of the IS is linked to its IaaS built in the cloud. The security and anonymity of patient information are segregated between patient's nominative data (hosted in PIST only) and their medical data (hosted in REDCap only), thus defining a security by design.

A datawarehouse was created downstream of REDCap to collect reorganised data from the production database. This DWH is used for data extractions and analyses. It enables collecting data from the French National Health Data Hub and exchanges data with the ERNs.

The RaDiCo IS is trans-institutional. It currently covers 180 RD reference centres, regardless of their location. Most importantly, it has been designed to be able to integrate many more cohorts and RD reference centres without modifying its IS. Moreover, it is also available for including RD registries and their patients’ follow-up.

The benefit of someone joining the platform is multiple. She/he benefits from the membership of the RD community that sustains the cohort, has access to the platform resources provided by the epidemiological team and shares the IS, participates to the analysis and discussions, is included in the scientific papers, is involved in ancillary studies, and may suggest complementary investigations.

In Europe, several scattered registries are available for different RDs, but they are siloed and fragmented, and often driven by industrial purposes rather than epidemiological objectives. A European platform (EU RD platform) has been set up to standardize data collection and data exchange [81]. It is linked to the European RD registry Infrastructure (ERDRI) which supports existing registries, and new registries as well, and renders RD registries data searchable and findable [82]. The EU RD Platform also offers a data repository, the Joint Research Centre EUROCAT Central Registry on congenital anomalies [83].

National information systems for RD cohorts with available data architecture are seldom. The CEMARA IS was proposed for the French RD centres since 2007 [84]. A review of RD registries gave no specific information on the architecture of a RD IS [85]. A more recent study has formulated recommendations and guidelines for improving the quality of RD registries including guidelines for information technology infrastructure [86]. In parallel, the Euro-NMD Registry Hub has proposed a new initiative to overcome data silos and empower patients with neuromuscular diseases [87]. It is dedicated to connecting several platforms for neuromuscular diseases linked through FAIR data points.

According to recent studies, costs related to RDs are very high [88]. In this respect, the sustainability of e-cohort platforms should be encouraged to improve RD research and care. Over 50% of the RaDiCo platform is financed by academic or industrial contracts, but institutional funding remains essential to its sustainability.

## Conclusion

RaDiCo provides an innovative platform for RDs, enabling the parallel management of 13 e-cohorts that currently include more than 8000 RD patients. The IS is based on a three-tenant architecture developed according to a cloud computing Infrastructure as a Service. Several modules have been created to ensure data quality and security. Interoperability and FAIR principles have been privileged. The platform can include new national or European RD cohorts, without modifying its architecture.

## Supplementary Information

Supplementary Material 1.

## Data Availability

The data of the cohorts are not available presently since they are still running, and primary objectives have not been analyzed yet. The datasets analyzed will be available from the PIs of the corresponding cohorts on reasonable request.

## Abbreviations

AD: Active Directory
BO: Back Office
BNDMR: (Banque nationale de données maladies rares) National Rare Disease Data Bank
CDISC: Clinical Data Interchange Standards Consortium
CGM: CompuGroup Medical Company
DWH: Datawarehouse
EGCS: Electronic Gateway Controller Service
EJP-RD: European Joint Program on RDs
ERDERA: European Rare Diseases Research Alliance
ERN: European Reference Networks
FAIR: Findability, Accessibility, Interoperability, Reusability
GUID: Global Unique Identifier
HDS: Health Data Hosting
IdMR: (Identifiant Maladie Rare) unique RD identifier
IaaS: Infrastructure as a Service
KPI: Key Performance Indicator
PI: Principal investigator
PIST: Patient Identification System and Translation
R: Language and environment for statistical computing and graphics
RACI: Responsible, Accountable, Consulted, Informed
RDHN: Rare Disease Healthcare Networks
RDs: Rare diseases
REDCap: Research Electronic Data Capture
SAS: Statistical analytical software
SOA: Service-oriented architecture
SOP: Standardized operating procedures
XML: Extensible Markup Language
