# Supplementary material for: The RaDiCo information system for rare disease cohorts
Source: Orphanet J Rare Dis. 2025 Apr 8;20:166. doi: 10.1186/s13023-025-03629-z (PMC11980265; doi:10.1186/s13023-025-03629-z)
Supplement: Supplementary file 1 — Supplementary Material 1. [file 13023_2025_3629_MOESM1_ESM.docx]

Table Supl.1 : Ethical agreements for the RDs cohorts according to the French law.

| **Cohorts**  **acronyms** | **Ethical Committee agreement  per cohort (agreement number)** | **Date of agreement** | **CCTIRS** | **Date of agreement** | **CNIL** | **Date of  agreement** |  |
| --- | --- | --- | --- | --- | --- | --- | --- |
| AC-ŒIL | CEEI (IRB00003888) | 08/09/2015 | CCTIRS | 07/01/2016 | CNIL | 01/08/2016 |  |
| AcoStill | CPP Ile de France I (IRB IORG0008367) | 02/04/2016 | CCTIRS | 12/05/2016 | MR001 | not appl. |  |
| COLPAC | CPP Ile de France I (IRB IORG0008367) | 12/07/2016 | CCTIRS | 19/05/2016 | MR001 | not appl. |  |
| DCP | CEEI (IRB00003888) | 06/10/2015 | CCTIRS | 12/11/2015 | CNIL | 04/10/2016 |  |
| ECYSCO | CEEI (IRB00003888) | 08/09/2015 | CCTIRS | 12/09/2015 | CNIL | 30/09/2016 |  |
| EURBIO Alport | CPP Ile de France I (IRB IORG0008367) | 31/03/2016 | CCTIRS | 11/02/2016 | CNIL | 13/03/2017 |  |
| FARD | CPP Ouest V | 04/07/2017 | not appl. | not appl. | MR003 | not appl. |  |
| GenIDA | CEEI (IRB00003888) | 15/11/2016 | not appl. | not appl. | CNIL | 27/11/2015 |  |
| IDMet | CPP Ile de France I (IRB IORG0008367) | 31/03/2016 | CCTIRS | 11/02/2016 | CNIL | 18/11/2016 |  |
| MPS | CPP Ile de France I (IRB IORG0008367) | 06/09/2016 | CCTIRS | 12/09/2016 | MR001 | not appl. |  |
| PID | CEEI (IRB00003888) | 06/09/2015 | CCTIRS | 17/03/2016 | CNIL | 03/11/2016 |  |
| PP | CPP Ile de France I (IRB IORG0008367) | 12/09/2017 | not appl. | not appl. | MR003 | not appl. |  |
| SEDVasc | CEEI (IRB00003888) | 08/09/2015 | CCTIRS | 12/11/2015 | CNIL | 15/06/2016 |  |
|  |  |  |  |  |  |  |  |

CEEI: Comité d’évaluation éthique de l’Inserm; CPP: Comité de Protection des Personnes; CCTIRS: Comité Consultatif pour le Traitement de l’Information en Recherche en Santé; CNIL: Commission Informatique et Libertés;
MR001: cf <https://www.cnil.fr/sites/default/files/atoms/files/mr-001.pdf> ;
MR003: cf <https://www.cnil.fr/sites/default/files/atoms/files/mr-003.pdf>;

not appl. : not applicable according to the new « Loi Jardé » in force as of Nov 18, 2016
